# Survival of *Salmonella enterica* in Aerated and Nonaerated Wastewaters from Dairy Lagoons

**DOI:** 10.3390/ijerph111111249

**Published:** 2014-10-29

**Authors:** Subbarao V. Ravva, Chester Z. Sarreal

**Affiliations:** Produce Safety and Microbiology Research Unit, United States Department of Agriculture, Agriculture Research Service, Western Regional Research Center, Albany, CA 94710, USA; E-Mail: chester.sarreal@ars.usda.gov

**Keywords:** *Salmonella*, aeration, dairy wastewater, aerators, manure, survival, decimal reduction times, * Salmonella* Enteritidis, *Salmonella* Montevideo, *Salmonella* Thompson

## Abstract

*Salmonella* is the most commonly identified foodborne pathogen in produce, meat and poultry. Cattle are known reservoirs of *Salmonella* and the pathogen excreted in feces ends up in manure flush lagoons. *Salmonella enterica* survival was monitored in wastewater from on-site holding lagoons equipped or not with circulating aerators at two dairies. All strains had poor survival rates and none proliferated in waters from aerated or settling lagoons*.* Populations of all three *Salmonella* serovars declined rapidly with decimal reduction times (D) of <2 days in aerated microcosms prepared from lagoon equipped with circulators. Populations of* Salmonella* decreased significantly in aerated microcosms (D = 4.2 d) compared to nonaerated waters (D = 7.4 d) and in summer (D = 3.4 d) compared to winter (D = 9.0 d). We propose holding the wastewater for sufficient decimal reduction cycles in lagoons to yield pathogen-free nutrient-rich water for crop irrigations and fertilization.

## 1. Introduction

An estimated 1.3 million cases of foodborne illnesses, resulting in over 15,000 hospitalizations and 550 deaths in the USA, are linked annually to infections caused by non-typhoidal *Salmonella* [1]. *Salmonella* is the most commonly identified pathogen in produce, poultry, pork, eggs and luncheon meat products [2]. The largest multi-state outbreak of the decade, that sickened 1535 people in 42 states in 2008, resulted from the consumption of jalapeño and serrano peppers contaminated with* Salmonella* Typhimurium [2]. Outbreaks are frequently associated with the consumption of contaminated food or drinking water that was exposed to pathogen-laden animal manure or irrigation water tainted with manure. Domestic livestock, such as dairy cows, are an established primary reservoir for human pathogenic bacteria [3]. One firewall in the prevention of foodborne illness outbreaks is to minimize the risk of on-farm manure contamination of food products. Designing effective and sustainable intervention strategies requires an understanding of pathogen prevalence and survival dynamics in farm environments.

Several studies have monitored on-farm shedding and prevalence of *Salmonella* [4,5,6]*,* and a link has been established between high livestock densities and prevalence [7]. In addition, nearly all dairies are likely to be positive for *Salmonella*, but in terms of numbers of animals shedding, a reasonably small percentage of farms appear to account for most *Salmonella*-positive cattle [6]. A high prevalence was also observed during summer months followed by fall and spring [7]. Furthermore, *Salmonella* was detected in waters, soil, cattle feces and wildlife in the vicinity of produce production areas [8,9]; in egg layer houses [10] and in fecal slurries from pig farms [11]. However, pathogen population dynamics in dairy wastewater remains largely unknown.

Environmental survival of *Salmonella* has been found to be varied and influenced by a number of factors. Destruction of *Salmonella* was observed with decimal reduction times (D-values) as fast as 10 min in diluted sludge from a thermophilic waste treatment plant [12], to a 7-year survival in soil from an outbreak-associated almond orchard [13]. The rate of destruction during storage of contaminated cattle and poultry manure was temperature-related [14,15]. The fastest decline was observed at increasing and still non-lethal temperatures ranging from 4 to 37 °C. A half-day D-value was reported for *Salmonella* Typhimurium at 37 °C in poultry manure, whereas a slow destruction with a D-value of 1 to 2 weeks was noticed at 4 °C [14]. Temperatures of 60 °C attained during composting of biowaste or garden wastes also eliminated *Salmonella* Senftenberg within 10 h [16] and in less than 3 days at 55 °C in dairy manure compost [17]. Conversely, mature biowaste compost supported longer survival (3 months) of *Salmonella* [18,19]. A simple intervention, which might result in pathogen reductions in stored manure, even at low temperatures, is aeration.

We evaluated earlier the influence of aeration on the survival of pathogenic *Escherichia* *coli* O157:H7 and observed that aeration did not enhance the destruction of the pathogen, but strain variations in survival were noticed under both aerated and non-aerated conditions [20]. Anaerobic digestion of pig slurry, in one study [21], appeared to enhance the destruction of *Salmonella* compared to aerated slurries. Thus, we determined the fate of outbreak strains of *Salmonella* in wastewater from dairy manure flush holding lagoons with and without circulating aerators to examine the impact of aeration. Controlled studies were conducted by inoculating microcosms containing both types of water. Microcosms prepared using wastewater from lagoons with installed aerators were aerated during the studies.

## 2. Experimental Section

### 2.1. Dairy Manure Flush Wastewater 

Aerated and non-aerated flush water was collected from manure lagoons from two medium-sized (~600–800 milking head) dairies (Oakdale, CA, USA). Dairy A uses aerators in its holding lagoons. In contrast, dairy B did not use aerators, but uses two sequential settling lagoons. Dairy A separates manure solids by holding the alley flush water for <24 h in temporary settling pit to allow the manure solids to settle. Water from these settling pits is then pumped into lagoons equipped with CirCulators^TM^ (Natural Aeration, Inc., Reardon, WA, USA). For additional details on circulating aerators see Ravva* et al.* [20] and Rumburg* et al.* [22]. 

Both dairies use the water from its lagoons for flushing the free-stall lanes, to irrigate, and fertilize pastures. The manure management practices followed by dairy B are typical to diaries in central valley of California. Chemical analysis (A & L Western Agricultural Labs. Inc., Modesto, CA, USA) of wastewaters was performed (Table 1).

**Table 1:** Chemical characteristics of wastewater from two dairies.

Chemical component ^a^	Summer Sampling ^b^			Winter Sampling ^c^			
	Dairy A-CirCulators	Dairy A-Settling Lagoon	Dairy B-Settling Lagoon 2	Dairy A-CirCulators	Dairy A-Settling Lagoon	Dairy B-Settling Lagoon 1	Dairy B-Settling Lagoon 2
N	278	318	512	1284	1476	1022	1035
P	89	82	53	67	105	107	130
K	465	395	363	695	813	811	481
S	67	30	7.0	69	90	36	37
Mg	90	95	102	129	158	120	126
Ca	96	123	140	255	332	182	194
Na	194	194	151	342	394	185	160
Fe	8.4	34	4.8	9.1	8.8	11	18
Al	3.6	5.1	1.7	5.6	7.9	9.8	5.4
Mn	0.3	0.8	0.6	1.5	2.3	1.3	1.3
Cu	<0.1	<0.1	<0.1	0.5	0.6	0.5	0.3
Zn	0.1	<0.1	<0.1	1.8	2.2	1.4	1.1
EC	3.8	4.2	4.9	8.0	9.3	5.6	4.9
OM	500	1500	1800	3873	5701	2298	2554
NO_3_^−^	<0.5	<0.5	<0.5	<0.5	0.6	<0.5	<0.5
NH_4_^+^	181	255	270	396	642	332	345
BOD	506	453	578	2163	2410	927	476
COD	1541	1785	1825	1980	2500	460	762
TSS	280	335	230	1867	2765	393	840
C:N ratio	1:1	3:1	2:1	2:1	2:1	1:1	1:1

Notes: **^a^** Values are in µg/mL except for electrical conductivity (EC), which is expressed as mS/cm; OM, organic matter; BOD, biological oxygen demand; COD, chemical oxygen demand; TSS, total suspended solids; **^b^** sampled July ‘02; **^c^** sampled January ‘03.

### 2.2. Isolation of Salmonella from Manure and Wastewaters 

One-hundred samples from dairies A and B were collected in summer and winter months. Samples included pooled fresh manure, wastewater from circulated and settling lagoons, and wet/dry manure from on-site manure piles. One mL or 1 g samples were inoculated into 9 mL of selective enrichment media. One hundred μL portions of serial dilutions of enrichments were plated on selective media for isolations. *Salmonella* was isolated after 24 h enrichments in Rappaport-Vassiliadis medium [23] (Sigma-Aldrich, St. Louis, MO, USA) followed by plating on SS agar (Becton, Dickinson and Co., Franklin Lakes, NJ, USA). Presumptive colonies were further characterized on brilliant green and triple sugar iron agars (Becton, Dickinson and Co.). Anti-*Salmonella* Dynabeads^®^ were used following the manufacturer’s instructions (Dynal Biotech Inc., Lake Success, NY, USA) for detection of low level pathogens by immuno-magnetic separation. The suspect organisms were characterized by API-20E for *Salmonella* and Biolog Microbial Characterization system (Biolog, Hayward, CA, USA). The identities were confirmed by 16S rRNA sequencing of 500 base pairs (Microbe Inotech Labs., Saint Louis, MO, USA) followed by sequence comparisons against MicroSeq database (Applied Biosystems, Foster City, CA, USA).

### 2.3. Fate of Salmonella in Wastewater 

Freshly collected wastewater acclimated overnight at room temperature was used to prepare the microcosms to monitor the fate of inoculated pathogens. Wastewater microcosms from dairy A and B were inoculated at ~10^7^ CFU/mL and monitored for pathogen decreases under both aerated and nonaerated conditions. The fate of *S. enterica* serovars (Table 2) was evaluated by establishing microcosms twice during summer (one week apart in July) and once during winter (January). 

**Table 2:** *Salmonella* strains.

Organism ^a^	Strain No.	Source	Details ^b^
*Salmonella enterica* serovar Enteritidis	MM155	Almond outbreak	RM2970; original isolation from soil drag swab, 10/01, LJH 620; PT30; University of California (UC), Davis
*S. enterica* serovar Montevideo	MM156	Almond outbreak	RM2977; LJH 627, UC, Davis
*S. enterica* serovar Thompson	MM157	Stool from 14 year old girl from Pennsylvania	RM2270; original source, CDC, the Salmonella Reference Laboratory

Notes: **^a^** All organisms were marked with rifampicin (110 μg/mL) and nalidixic acid (50 μg/mL) resistance; **^b^** RM strains were from Produce Safety Microbiology and Research Unit, Albany, CA, USA.

The tests were conducted in 1 L microcosms in 2-L Nalgene magnetic culture vessel fitted with stir bar suspended from the closure (Nalgene Nunc International, Rochester, NY, USA). Microcosms established using wastewater from lagoons fitted with CirCulators were kept aerated by stir bars during the tests while water from noncirculated settling lagoons was used to produce nonaerated microcosms. The microcosms were incubated at 25 ± 1 °C for 4 to 6 weeks. The winter test was conducted in triplicate, whereas the summer tests were not replicated but repeated twice. Uninoculated controls to monitor native *Salmonella* were included. Samples were collected at various intervals and analyzed for *Salmonella*.

The temperature of the microcosms was monitored continuously with an immersion probe and a Dickson FT121 electronic recorder (Dickson, Addison, IL, USA). Dissolved oxygen was measured with an YSI 5100 oxygen meter equipped with a 5010 self-stirring BOD probe (YSI, Inc., Yellow Springs, OH, USA), and pH was monitored by using an Accumet AP63 pH meter (Accumet-Fisher, Hampton, NH, USA).

### 2.4. Monitoring Salmonella 

The strains of *Salmonella* (Table 1) used were marked with spontaneous rifampicin and nalidixic acid resistance to facilitate enumeration from wastewater. Salmonellae were monitored by plating 100 μL portions of serial dilutions of wastewater in phosphate buffered saline (0.01 M, pH 7.4) on Luria-Bertani (LB) agar supplemented with rifampicin (100 μg/mL), nalidixic acid (50 μg/mL) and cycloheximide (50 μg/mL). The nalidixic acid marker was essential in differentiating native *rif* resistant colonies that are slightly smaller but indistinguishable in color from the test strains. Cycloheximide was used to control the growth of fungi. The limit of detection from undiluted wastewater was improved by plating triplicate 100 μL samples and pooling the counts from all three. Enumeration plates were incubated overnight at 37 °C. Salmonellae from un-inoculated wastewater were monitored as described previously.

### 2.5. Statistical analysis. 

The pathogen decline numbers from each microcosm were transformed logarithmically and the days required for one log decline (D) were calculated from linear regressions. Analysis variance tests were performed to determine the influence of aeration, strain variations, and season on survival of salmonellae in wastewater microcosms. Analysis of variance for D was performed at *p* > 0.05 for normality and equal variance tests (SigmaPlot 11, SyStat Software, Inc., San Jose, CA, USA) and the test of significance was set at *p* = 0.05.

## 3. Results

### 3.1. Chemistry of Wastewaters 

Chemical composition of wastewaters indicated that waters collected during the winter month had higher values in almost every parameter compared to summer collection from both dairies (Table 1). The chemical component values for wastewater from dairy A settling lagoon were higher compared to aerated lagoons on both sampling periods. The water chemistry of sequential settling lagoons for dairy B during winter was unexpectedly similar although the solids would have settled in lagoon 1 prior to reaching the second lagoon.

### 3.2. Salmonella in Wastewater from Dairies 

Salmonellae were not found in fresh or dry manure, or wastewater samples from both dairies. Many colonies with suspect coloration and the morphology of *Salmonella* were detected on SS agar and brilliant green agar plates. All suspects were negative by API 20 E for *Salmonella*. Two of the suspects from API 20 E were characterized as *Citrobacter youngae* and *Proteus mirabilis* by 16S rRNA gene sequencing. All other suspects characterized by API 20 E also belong to either genus *Citrobacter* or *Proteus*. Efforts to isolate *Salmonella* from wastewater by immuno-magnetic separation from selective enrichments followed by plating on selective media were also negative.

### 3.3. Survival of Salmonella in Wastewater 

**Figure 1:**
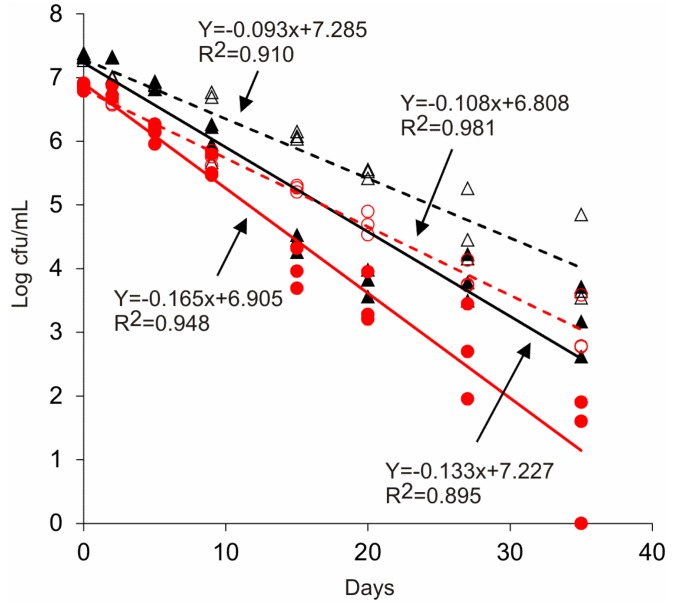
Kinetics of survival of *Salmonella enterica* in wastewaters from dairy A with and without CirCulators. Survival of serovars Montevideo (black filled triangle-aerated; black open triangle-nonaerated) and Thompson (red filled circle-aerated; red open circle-nonaerated) is shown.

*S. enterica* serovars declined rapidly from circulated water of dairy A (Table 3, Table 4 and Table 5) compared to waters from settling ponds from both dairies. The studies were repeated three times during summer and winter months to confirm the enhanced decreases in circulated water from dairy A aerated in microcosms. On every occasion, *Salmonella* serovars declined significantly in circulated wastewater (mean D-value for aeration = 4.2 d) as compared to settling waters from both dairies (mean D-value 7.4, Table 4). Individual D values ranged between 1.4 to 11.1 d (Table 3). Serovar Enteritidis declined fastest from aerated water from dairy A (Table 3), while Montevideo survived longest in nonaerated settling lagoon waters from both dairies (Table 3 and Table 4). Overall, Enteritidis populations declined significantly (D = 3.8 d; Table 4) in aerated and non-aerated waters from both dairies. In both tests during July, all 3 serovars disappeared in <8 d from aerated water of dairy A. In January, high populations (10^4^ CFU/mL) of Montevideo and Thompson were detected at the termination (35 d; Figure 1) of settling water microcosms from both diaries. *Salmonella* was not detected from uninoculated microcosms. There was a significant seasonal influence on the survival of *Salmonella*. Irrespective of serovars, least square mean D-values for summer and winter were 3.4 and 9.0 days, respectively. The values are significantly different at p < 0.001. Average daily temperatures during the months of July ‘02 and January ‘03 were 25.9 ± 2.4 °C and 9.4 ± 2.2 °C, respectively [24]. In addition, decreases in salmonellae from nonaerated water collected from the second sequential settling lagoon from dairy B were similar to aerated water from dairy A (Table 5), although the levels of organic matter and total suspended solids were substantially different. Overall, there were significant aeration and seasonal effects, but chemical composition of wastewaters did not influence the survival of *Salmonella*. Temperature measured in microcosms was 25.7 ± 0.2 °C. Dissolved oxygen and pH of the microcosms during the incubations were, 2.0 ± 0.2 mg/L and 8.04 ± 0.02 for aerated treatments and 0.05 ± 0.02 mg/L and 7.47 ± 0.04 for non-aerated microcosms, respectively.

**Table 3:** Range of D-values obtained for *Salmonella* strains from microcosm tests using wastewaters from two dairies.

*S. enterica* Serovars	D-value ^a^ (Fastest/Slowest)	Wastewater Source
Enteritidis	1.4	Circulated—dairy A
	6.1	Settling—dairy A
Montevideo	1.7	Circulated—dairy A
	11.1	Settling lagoon 1—dairy B
Thompson	1.8	Circulated—dairy A
	9.4	Settling—dairy A

Notes: ^**a**^ Fastest or slowest decimal reductions obtained in three tests. Corresponding sources of water from which extreme D-values obtained are shown.

**Table 4:** Influence of aeration on survival of *Salmonella enterica* in dairy wastewater.

Serovar ^a^	Strain	D-value, d		Mean	*p-*value	α at 0.05
		Aerated	Not Aerated	(Serovars)		
Enteritidis	MM155	2.3 b	5.3 b	3.8	0.084	0.316
Montevideo	MM156	5.4 a	9.0 a	7.2		
Thompson	MM157	4.8 ab	8.0 a	6.4		
Mean (aeration)		4.2 B	7.4 A			
*p*-value		0.005				
α at 0.05		0.793				

Notes: **^a^** Data from three repeat experiments were used for 2-way ANOVA (total dF = 35). Wastewaters from dairies A and B were compared. Numbers followed by the same letter within the same group are not significantly different.

**Table 5:** Effect of aeration in microcosms prepared from wastewater from two dairies with different manure management practices on survival of *Salmonella enterica* serovars.

Manure Water Source	Aeration in Microcosms	D-value, Days ^a^		Mean (Water Source)	α at 0.05	*p*-value
		Montevideo	Thompson			
Dairy A—CirCulators	Aerated	7.6 b	6.1 b	6.9 C	0.987	<0.001
Dairy A—settling lagoon	Nonaerated	11.0 a	9.4 a	10.2 A		
Dairy B—settling lagoon 1	Nonaerated	11.1 a	9.4 a	10.2 A		
Dairy B—settling lagoon 2	Nonaerated	8.2 b	7.9 ab	8.0 B		
Mean (serovar)		9.5 A	8.2 B			
α at 0.05		0.552				
*p*-value		0.026				

Notes: ^a^ Two way ANOVA. Total dF = 23. Serovar Enteritidis was not included in this January study. Numbers followed by the same letter within each group are not significantly different.

## 4. Discussion

Pathogenic bacterial contamination of dairy, meat, fruit and vegetable products is known to be caused by direct or indirect exposure to animal waste [3]. Finding the on-farm sites of pathogen proliferation will facilitate developing effective intervention strategies. Salmonellae were not found in manure and wastewater samples from two different dairies. A low prevalence of 2.3% was achieved in an earlier study based on extensive sampling (2401 samples) of soil/sediment, water, cattle feces, wildlife and produce [9]. However, only one out of 795 fecal samples tested positive. Thus, prevalence of salmonellae in fecal samples from dairies and feedlots appears to be extremely low to none.

The rapid decline in populations of all *Salmonella* serovars in aerated wastewater with a D-value of 1.6 ± 0.2 d (Table 3) is noteworthy. A similarly rapid destruction of *Salmonella* Typhimurium in slurries prepared from poultry and cow manures [14,15] was reported, but those tests were done at a higher temperature of 37 °C. Even more rapid killing with a D-value of 10 min at a much higher temperature of 50 °C was reported for *S. enterica* in sludge from a thermophilic waste treatment plant [12]. Other reports of rapid decreases were reported for *Salmonella* Typhimurium in sewage sludge [25], in paper mill effluents [26] and in manure or slurries from pigs, sheep, cattle or poultry applied on fescue plots [27]. The rapid decreases of salmonellae from manure impacted and other environments appear to be the reason for failure to find *Salmonella* in manure and wastewater samples from dairies.

There is a significant difference in survival of salmonellae in aerated wastewaters compared to non-aerated settling lagoon waters (Table 4 and Table 5). These results are in contrast to our previous study where survival of *E. co*li O157:H7 was not influenced by aeration [20]. The rapid killing of salmonellae in aerated microcosms doesn’t appear to be related to the chemistry of wastewaters (Table 1) but an increase in pH (from 7.5 to 8.0) along with a significant increase in dissolved oxygen level (from 0 to 2 mg/L) was observed with aeration. A similar increase in pH along with significant decreases in populations of *Salmonella* Typhimurium in chicken manure was linked to ammonia release [28]. Alternatively, salmonellae can be killed with ammonia released by raising the pH of the manure [29]. Furthermore, we found that both basic and acidic organic components in wastewaters are inhibitory to *E. coli* O157:H7 [30] and it is likely that an increase in pH brings acidic compounds in to solution and possibly inhibit salmonellae. In addition, aeration enhances the growth of native aerobic microorganisms that are likely to compete with salmonellae for nutrients [20,30]. Increases in native bacteria also facilitate enhanced predation, and protozoa native to wastewaters [31] and other environments are known predators of *E. coli* O157:H7 and salmonellae [32,33]. Thus, aeration brings about both direct and indirect effects in inhibiting the growth of salmonellae.

Salmonella populations decreased significantly from wastewaters collected during the summer. We observed, in a previous study, a similarly rapid decrease of *E. coli* O157:H7 populations in wastewaters collected during summer months as opposed to winter [20]. D-values comparable to the winter study were also obtained for *Salmonella* inoculated in 35,000 L volumes of cattle slurries incubated either in summer at a temperature of 12.4 °C or in winter at a temperature of 4.3 °C [34]. Since microcosms were tested at 25 °C in this study, it appears that seasonal fluctuations in temperature have an indirect effect on the chemistry and biology of wastewaters. Increased growth of competing organisms during warm summer as one reason for rapid decreases of salmonellae, and we found earlier that the elimination of competition facilitates proliferation of the introduced pathogen [30].

The rapid reduction rates observed for salmonellae in wastewaters are uncommon in other environments, and in one example *Salmonella* Enteritidis persisted for nearly seven years in an almond orchard soil [13]. A cycle of transmission, recontamination and regrowth in wet almond hulls was postulated for such persistence. In an another case, *Salmonella* Newport persisted for nearly an year in manure-amended soil [35]. Favorable environmental temperatures [15,20], readily available nutrients [30,36] and adequate moisture levels [13] are some factors that promote pathogen regrowth and persistence. Breaking the cycle of transmission and regrowth is critical for any successful on-farm intervention strategies. Some strategies for reducing salmonellae from dairies (and feedlots) that require further scrutiny are: retaining wastewater in lagoons for enough decimal reduction cycles [20], pH adjustment [29] and composting [37,38].

## 5. Conclusions

Outbreak-related isolates of *Salmonella* Enteritidis, *Salmonella* Thompson and *Salmonella* Montevideo decreased rapidly in numbers from wastewater from dairy lagoons. All three pathogens decreased significantly in aerated microcosms prepared with wastewater from lagoon equipped with circulating aerators in comparison to nonaerated microcosms. As the mean decimal reduction times for salmonellae reach less than 8 days in wastewaters, retaining the water for sufficient reduction cycles in lagoons should yield nutrient-rich pathogen-free water for crop irrigations and fertilization.
